# Dr. Lewis Arthur Welles Alleman

**Published:** 1918-01

**Authors:** 


**Dr. Lewis Arthur Welles Alleman**, Geneva, N. Y. Jefferson Medical College, 1886, age 55, died November 3.

